# Characterization of plasma lipidomics in adolescent subjects with increased risk for type 1 diabetes in the DiPiS cohort

**DOI:** 10.1007/s11306-020-01730-x

**Published:** 2020-10-08

**Authors:** Agnes Andersson Svärd, Simranjeet Kaur, Kajetan Trôst, Tommi Suvitaival, Åke Lernmark, Marlena Maziarz, Flemming Pociot, Anne Julie Overgaard, The DiPiS Study Group

**Affiliations:** 1 Department of Clinical Sciences, Skåne University Hospital, Lund University/CRC, Malmö, Sweden; 2 Steno Diabetes Center Copenhagen, Niels Steensens Vej 2, Gentofte, Denmark; 3 Department of Clinical Medicine, Faculty of Health and Medical Sciences, University of Copenhagen, Copenhagen, Denmark

**Keywords:** Type 1 diabetes, Lipidomics, Metabolomics, Autoantibodies, Autoimmunity, Prediction

## Abstract

**Introduction:**

Type 1 diabetes (T1D) is caused by the destruction of pancreatic islet beta cells resulting in total loss of insulin production. Recent studies have suggested that the destruction may be interrelated to plasma lipids.

**Objectives:**

Specific lipids have previously been shown to be decreased in children who develop T1D before four years of age. Disturbances of plasma lipids prior to clinical diagnosis of diabetes, if true, may provide a novel way to improve prediction, and monitor disease progression.

**Methods:**

A lipidomic approach was utilized to analyze plasma from 67 healthy adolescent subjects (10–15 years of age) with or without islet autoantibodies but all with increased genetic risk for T1D. The study subjects were enrolled at birth in the Diabetes Prediction in Skåne (DiPiS) study and after 10–15 years of follow-up we performed the present cross-sectional analysis. HLA-DRB345, -DRB1, -DQA1, -DQB1, -DPA1 and -DPB1 genotypes were determined using next generation sequencing. Lipidomic profiles were determined using ultra-high-performance liquid chromatography quadrupole time-of-flight mass spectrometry. Lipidomics data were analyzed according to genotype.

**Results:**

Variation in levels of several specific phospholipid species were related to level of autoimmunity but not development of T1D. Five glycosylated ceramides were increased in insulin autoantibody (IAA) positive adolescent subjects compared to adolescent subjects without this autoantibody. Additionally, HLA genotypes seemed to influence levels of long chain triacylglycerol (TG).

**Conclusion:**

Lipidomic profiling of adolescent subjects in high risk of T1D may improve sub-phenotyping in this high risk population.

**Electronic supplementary material:**

The online version of this article (10.1007/s11306-020-01730-x) contains supplementary material, which is available to authorized users.

## Introduction

Type 1 diabetes (T1D) is an autoimmune disorder caused by destruction of the pancreatic islet beta cells resulting in total loss of insulin production (Insel et al. 2015; Katsarou et al. 2017). Lifelong treatment with daily injections of insulin is needed to sustain life. T1D is strongly associated with HLA-DR-DQ. Circulating islet autoantibodies are robust biomarkers for beta-cell autoimmunity and increased risk for progression to clinical onset of T1D (Krischer et al. 2015, 2017a, b; Verge et al. 1996; Ziegler et al. 2013). Presence of islet autoantibodies may precede clinical onset months to years (Ilonen et al. 2013; Ziegler and Nepom 2010; Ziegler et al. 2013).

Studies have shown that a faster progression to clinical T1D is related to the number of islet autoantibodies (Knip et al. 2010; Verge et al. 1996; Ziegler et al. 2013). As in the previously described staging of T1D (Insel et al. 2015), progression from multiple autoantibodies (Stage 1) to clinical onset T1D (Stage 3) may also be affected by genetic, primarily non-HLA genetic factors, in addition to environmental factors (Söderström et al. 2012; Stene et al. 2010). It is still unknown how islet autoimmunity is triggered and why some children progress to T1D and others do not.

Children who develop T1D before the age of 4 years have previously been found to have significantly less phospholipids at birth than healthy controls (La Torre et al. 2013). Another study investigated differences in serum from children followed from birth until clinical T1D and healthy controls and found that a distinct cord blood lipidomic profile characterized T1D progressors, a molecular signature of seven lipids predicted high risk for progression to T1D (Oresic et al. 2013). If altered blood lipid composition reflects increased risk for T1D, lipidomics may provide a new mean for identifying biomarkers for progression to islet autoimmunity and T1D. Here we investigate lipidomic profiles in adolescent subjects with a high-risk HLA genotype who have not progressed to T1D and if lipid patterns could be tied to the HLA genotypes. We further investigate if there are any differences in plasma lipidomic composition in relation to number of islet autoantibodies known to be associated with an increased risk for T1D.

## Materials and methods

### Study participants

**Fig. 1:**
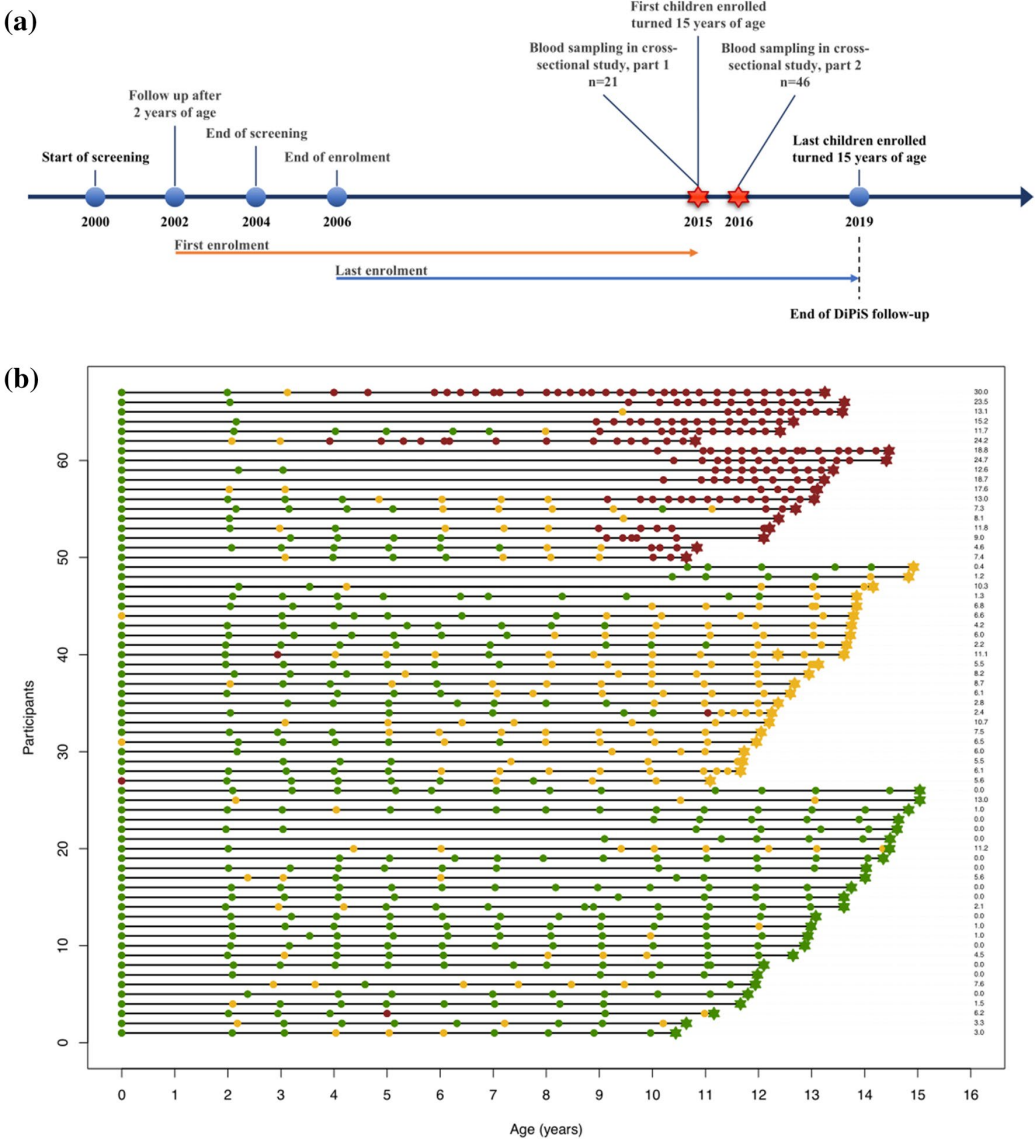
Schematic of the Diabetes Prediction in Skåne (DiPiS) study timeline and time of sampling. **a** Schematic of the Diabetes Prediction in Skåne (DiPiS) study timeline and cross-sectional study sampling that outline key events of enrolment and follow-up in DiPiS and sampling in the present study. Children with increased risk of T1D were followed from 2 years of age either annually (none or one autoantibody) or every 3 months (if two or more islet autoantibodies were detected at any earlier visit) until the age of 15 or diagnosis of T1D. The sampling into our study was divided in two parts, part 1 where n = 21 subjects were sampled and part 2 where n = 46 subjects were sampled. **b** The timeline plot shows the visits (circles for visits as part of DiPiS follow-up, stars for time of sampling into our study) and autoantibody count (0 = green, 1 = yellow, 2+ = red). During follow-up, 13 children have never tested positive for an autoantibody, 33 tested positive for 1 autoantibody at least once during follow-up, and 21 tested positive for multiple autoantibodies at least once. Autoimmunity burden, measured as area under the curve of autoantibodies during DiPiS follow-up, is presented to the right (Color figure online)

Blood samples were collected from adolescent subjects (n = 67), aged 10–15 years, at increased risk of T1D enrolled in the Diabetes Prediction in Skåne (DiPiS) study (Larsson et al. 2005; Lundgren et al. 2014) as previously described (Andersson Svärd et al. 2020) (Table 1). DiPiS is a prospective population-based study, following children from birth with the aim to investigate genetic and environmental factors that may contribute to the development of T1D (Elding Larsson 2016). Briefly, individuals with either T1D high-risk HLA defined by HLA-DR-DQ genotypes, a first degree relative with T1D, islet autoantibodies detected in cord blood, or a mother with gestational diabetes (Larsson et al. 2004, 2005) were identified by screening newborn children (n = 35,000). Children (n = 03,889) with increased risk of T1D were followed either annually (none or 1 autoantibody) or every third month (2 or more autoantibodies) from 2 years of age until 15 years of age or diagnosis of diabetes, whichever occurred first. The eligibility criteria for enrolment in the present study was high-risk HLA-DQ. Out of 98 DiPiS participants who were asked to participate, 67 (68%) subjects agreed to participate (Table 1). A schematic representation of the DiPiS timeline and cross-sectional study sampling is presented in Fig. 1. The participants were not instructed to fast before blood draw. The participants donated blood to the present study during their annual DiPiS visit. Originally this study cohort was obtained primarily to test the hypothesis that the number of islet autoantibodies affect cell surface HLA-DQ expression on blood leukocytes (Andersson Svärd et al. 2020).

**Table 1:** Children (n = 67) at an increased risk of T1D, stratified by the number of islet autoantibodies detected at cross-sectional sampling, that have been followed in the Diabetes Prediction Study in Skåne (DiPiS)

Study subjects (n = 67)	No autoantibodies (n = 26)	One autoantibody (n = 23)	Multiple autoantibodies (n = 18)	p value
Females, n (%)	14 (53.8)	11 (47.8)	10. (55.6)	0.867
Age (years), mean (sd)	13.18 (1.38)	12.98 (1.07)	12.72 (1.11)	0.459
Autoimmunity burden, mean (sd)	2.34 (3.63)	5.73 (3.01)	15.08 (7.09)	4.54E−9
HLA-DQ2/8, n (%)	25 (96.2)	12 (52.2)	4 (22.2)	3E−6
Medium levels (interquartile ranges) of autoantibody titers (U/mL) at sampling				
GADA	12.31 (7.60–14.69)	278.75 (38.81–259.91)	436.07 (50.35–736.36)	
IA-2A	1.39 (0.77–1.73)	1.71 (1.09–1.81)	1260.81 (18.21–951.00)	
IAA	0.14 (0.00–0.17)	12.18 (0.00–0.32)	20.97 (0.34–3.56)	
ZnT8RA	13.83 (8.85–16.42)	15.30 (10.15–16.81)	243.63 (17.37–117.23)	
ZnT8WA	15.46 (8.39–21.75)	14.91 (7.61–19.87)	102.25 (3.92–78.23)	
ZnT8QA	14.61 (7.06–21.55)	19.55 (10.61–19.55)	169.78 (16.16–115.93)	

Autoimmunity burden is measured as area under the curve of islet autoantibodies during DiPiS follow-up. Variances between groups were assessed for age at sampling (One-Way ANOVA), gender distribution (Chi-square test), autoimmunity burden (Kruskal–Wallis test) and high HLA risk (HLA-DQ2/8). P values were corrected for multiple comparisons using the Benjamini–Hochberg method. P values were considered significant < 0.05 and all statistical analysis were performed in SPSS from IBM. Age at sampling and gender were not significant. Autoimmunity burden and HLA-DQ2/8 genotype were presumed to vary between groups

The current cohort represent subjects at various stages of the pathogenesis (Andersson Svärd et al. 2020; Insel et al. 2015). Blood glucose and HbA1c were available for all participants with intense follow-up. The measurements were normal (p-glucose < 11.1 mmol/L and HbA1c ranged between 27 and 40 mmol/mol American Diabetes 2018) and the children with two or more islet autoantibodies would belong to Stage 1 in the current nomenclature (Insel et al. 2015).

### Islet autoantibodies

Autoantibodies against insulin (IAA), glutamate decarboxylase (GADA), insulinoma-associated protein-2 (IA-2A) and three variants of Zinc Transporter 8 (ZnT8A) against arginine, tryptophan or glutamine at position 325 (R/W/Q, respectively) were analyzed in plasma using in-house methods. The autoantibodies have been measured annually or quarterly in sera or plasma throughout the DiPiS study as well as our cross-sectional sample (Fig. 1b) as previously described (Andersson Svärd et al. 2020). Autoantibodies in the cross-sectional samples were analyzed in plasma from blood diluted 1:2 in RPMI1640 for n = 24 subjects as well as both in plasma from undiluted blood and plasma from blood diluted 1:2 in RPMI1640 for n = 43 subjects.

Intra-assay and inter-assay coefficients of variation, respectively, in the cross-sectional samples were 6.9% and 8.5% for GADA, 9.8% and 6.4% for IA-2A, 10.0% and 11.6% for screening of IAA, 7.4% and 6.8% for IAA COMP, 9.8% and 1.2% for ZnT8RA, 9.8% and 4.4% for ZnT8WA, and 9.6% and 5.0% for ZnT8QA.

Islet Autoantibody Standardization Program (IASP) help evaluate and harmonize laboratory autoantibody assays. Our laboratory is part of IASP and at the 2018 serum exchange, sensitivity and specificity was set at 64.0% and 94.5%, respectively, for GADA; 62.0% and 100.0%, respectively, for IA-2A; 18.0% and 96.7%, respectively, for IAA; 40.0% and 100.0%, respectively, for ZnT8RA; 54.0% and 100.0%, respectively, for ZnT8WA; as well as 52.0% and 100.0%, respectively, for ZnT8QA.

### HLA next generation sequencing

Dried blood spot punch-outs (6 mm) were sent blinded to Cisco Systems, Inc. (Seattle, USA) to perform Next Generation Sequencing (NGS) of HLA Class II -DRB345, -DRB1, -DQA1, -DQB1, -DPA1 and -DPB1. Briefly, PCR-based HLA amplification and sequencing with Illumina MiSeq technology is used in HLA NGS as previously described (Zhao et al. 2016). Extended HLA haplotypes were assembled with help from allelic information and an online database (Allele Frequencies in Worldwide Population, http://www.allelefrequencies.net) (Kempson et al. 2014).

### Lipidomics

Lipidomic profiling was performed on plasma samples originating from whole blood samples diluted 1:2 in RPMI1640 media before isolation of plasma. The dilution of whole blood with media did not influence the lipid extraction product as evaluated by pilot studies (data not shown).

Extraction of lipids were performed by the following procedure: 10 µL of plasma sample, technical quality control sample and pools of plasma, and standards were added 10 µL of 0.9% NaCl, 28 µL of a standard mix containing 14 different synthetic lipids [lipid standard mix contained: 1.000 µg/mL PE (17:0/17:0), 0.850 µg/mL SM (d18:1/17:0), 0.995 µg/mL Cer (d18:1/17:0), 1.215 µg/mL PC (17:0/17:0), 1.022 µg/mL LPC (17:0), 1.000 µg/mL PC (14:0/d13), 1.000 µg/mL TG (16:0/16:0/16:0)-13C3, 1.000 µg/mL TG (8:0/8:0/8:0)-13C3, 1.000 µg/mL PC (16:0/d31/18:1), 1.006 µg/mL PG (17:0/17:0), 1.003 µg/mL PS (17:0/17:0), 1.091 µg/mL PA (17:0/17:0), 1.067 µg/mL TG (15:0/15:0/15:0), 1.281 µg/mL TG (19:0/19:0/19:0)] and 92 µL of chloroform:methanol (2:1, v/v) and were vortexed and left incubating on ice for 30 min. Phases were separated by centrifuging the samples for 3 min at 10,000 rpm at 4 °C and subsequently 60 µL of the lower phase were transferred by first pipetting 60 µL of chloroform:methanol (2:1, v/v) into HPLC glass vials with glass inserts, and using the same tip, transferring the lower phase from the samples to the glass vials. The order of the samples was randomized before the LC–MS analysis.

Lipid extracts were analyzed using an ultra-high-performance liquid chromatography quadrupole time-of-flight mass spectrometer (UHPLC-Q-TOF-MS) operated in the positive ion mode. The column was an Acquity UPLC™ BEH C_18_ 2.1 × 100 mm with 1.7 µm particles from Waters (Milford, CT, USA). Column temperature was kept at 50 °C and the temperature of the autosampler was set at 10 °C. The binary solvent system consisted of A: water (1% 1 M NH_4_Ac, 0.1% HCOOH) and B: LC–MS grade isopropanol∶acetonitrile (1∶1, 1% 1 M NH_4_Ac, 0.1% HCOOH). The gradient started from 65% A/35% B and reached 80% B in 2 min, 100% B in 7 min and remained at this level for next 7 min. The total run time, including a 7 min re-equilibration step, was 20 min. The flow rate was 400 µL/min and the injection volume of each sample 1 µL. The mass spectrometer was a 6550 iFunnel quadrupole time of flight from Agilent technologies (Agilent) interfaced with a dual jet stream electrospray ion source.

The acquisition mass range was m/z 100–1700 and the instrument was run using the extended dynamic range with an approximate resolution of 30,000 FWHM measured at m/z 1521.9715 (which is included in the tune mixture) during calibration of the instrument. Data were acquired using the MassHunters B.06.01 (Agilent) (Lankinen et al. 2011).

### Data analysis

#### Lipidomics

The open source software processing tool MZmine 2.21 was used to process the data obtained from the lipidomic analysis. Features in the spectra were annotated based on the internal spectral library and the LipidMaps online database. Features originating from the internal standards were detected in a targeted way based on the standard runs. Other features in the samples were processed by the following procedure: first the raw data was imported and next reduced using a crop filter resulting in a copy of the raw data file only including scans in the m/z range of 200–1000 and retention time range of 2.4–13.6 min. Mass detection was performed with the noise minimum intensity level limit set to 2.3E3. Chromatogram building was achieved using a minimum time span of 0.06 min between ions, minimum intensity of the highest data point of 6E3, and maximum difference between data points of m/z 0.005 (or 5 ppm).

The Local minimum search method was used for chromatogram deconvolution with, chromatogram threshold = 70%, minimum retention time range = 0.05 min, minimum relative height = 5%, minimum absolute height = 7.5E3, minimum ratio of peak top/edge = 1, and peak duration range = 0.06–1.0 min. Chromatograms deisotoping was performed using the isotopic peak grouper algorithm with the settings of m/z tolerance of 0.001 (or 10 ppm), and RT tolerance of 0.05 min, with most intense ion kept. The peak list was filtered to exclude false signals by using the settings for peak detection requiring 8–200 data points, 0–2 FWHM, a tailing factor between 0.36 and 2 and an asymmetry factor between 0.33 and 3. Subsequently, the peak list was row filtered for removing all rows which did not meet the requirement of minimum peaks in a row = 1. Peak alignment was achieved using join aligner method [m/z tolerance 0.006 (or 10.0 ppm), weight for m/z = 2, absolute RT tolerance = 0.2 min], absolute RT tolerance of 1 min, and a threshold value of 1. The peak list was row filtered again with minimum peaks in a row to 50% of total sample number. The peak list was afterwards gap-filled with the same RT and m/z range gap filler (m/z tolerance at 0.005 or 5 ppm) and was again row filtered. Finally, the peak list was annotated using internal library and the online database LipidMaps (https://www.lipidmaps.org/ ) with an m/z tolerance of 0.002 m/z or 10.0 ppm and a RT tolerance of 0.1 min. The annotation of features was based on equivalent injected standards (level 1) and structure information obtained previously with MS2 (level 2) (Sumner et al. 2007). The quality of all peaks that were successfully annotated based on the internal library were manually inspected. Poor quality features and features without annotation were discarded for further analysis.

#### Normalization of lipid data

The final peak list with annotations was imported into the free statistical environment R (R Development Core Team 2010) and the peak areas of the features were normalized to the internal standard with the highest correlation with the feature in question. The matched internal standard was used for normalizing the feature with the aim of removing technical variation from the measurements. Percentage relative standard deviation (%RSDs) in pooled study samples was calculated for peak areas for each identified lipid and a threshold of 20% was used.

#### Nomenclature and annotation of lipids

In this study we used the lipid convention outlined by the Lipid Maps Consortium. Lipids containing two fatty acid chains without further characterization are expressed as the sum composition of carbon atoms and double bonds [e.g. PC (38:6)]. Where acyl chains have been determined and the position is known, a forward-slash between acyl-chains is used [i.e. PC (16:0/22:6)]. The same nomenclature is used for other lipid classes and subclasses. Lipid species that were separated chromatographically were labelled with an ‘a’ or ‘b’ and describes the elution order. The use of ‘d’ in the notation of the sphingomyelins refers to 1,3 dihydroxy, and the E/Z designation in the triglycerides is used to define double-bond geometry.

The lipids detected in this study was annotated according to MSI level 2 (Members et al. 2007) by using internal standards added to the samples and based on mass according to the LipidMaps database.

### Statistical analyses

Variation in clinical characteristics were assessed between groups (Table 1) using One-Way ANOVA, Kruskal–Wallis or Chi-square test in SPSS from IBM. Variation in lipid levels according to number of positivity for different numbers of autoantibodies or specificity of autoantibody were evaluated using Students t-test or ANOVA in R. P values were corrected for multiple testing using the Benjamini–Hochberg (BH) approach (1995). P values < 0.05 were considered significant.

We performed hierarchical cluster analysis to group patients based on their HLA profiles and haplotypes. A dissimilarity matrix was created by computing all the pairwise dissimilarities (distances) between the individual data points based on “Gower’s distance” within R package “cluster”. The dissimilarity matrix was used as input for hierarchical cluster analysis using agglomeration complete linkage method in “hclust” function in R. Agglomerative clustering initially starts with n clusters, where n is the number of observations, assuming that each of them is its own separate cluster. Then the algorithm identifies the most similar clusters using the complete linkage method in hclust and groups them into larger clusters. This process was repeated until four clusters were identified. The cluster statistics were assessed using “cstats” function in R.

## Results

### Demographic characteristics of healthy subjects with and without islet autoantibodies

The research subjects included in this study all have an increased genetic risk for T1D. Variances between groups in numbers of islet autoantibodies were assessed for age at sampling (One-Way ANOVA), gender distribution (Chi-square test), autoimmunity burden (Kruskal–Wallis test) and high risk HLA (HLA-DQ2/8) (Chi-square test). Age at sampling and gender did not differ between the groups of no, single and multiple autoantibodies (Table 1). Autoimmunity burden, measured as area under the curve of autoantibodies during DiPiS follow-up, and HLA-DQ2/8 genotype varied significantly between the three groups of subjects. At the time of sampling, 13 of the subjects had never had detectable autoantibodies at any of the times measured.

To date, only 5 subjects (participant 50, 58, 64, 65, 66 in Fig. 1) have progressed to T1D after the cross-sectional sampling in the present investigation. These five subjects who were diagnosed with T1D 6–26 months after the cross-sectional sampling did not differ in lipid composition compared to the other subjects with multiple autoantibodies at sampling.

### Identification and quantification of lipids in plasma

The lipidomic analysis detected 128 different lipid features that were annotated based on the internal standard. The detected lipids were members of the following lipid classes: Sphingomyelin (SM), lysophosphatidylcholine (LPC), phosphatidylcholine (PC), phosphatidylethanolamine (PE), alkylphosphatidylcholine [PC(O)], alkenylphosphatidylcholine [PC(P)], alkylphosphatidylethanolamine [PE(O)] or phosphatidylethanolamine [PE(P)], phosphatidylinositol (PI), cholesterol ester (CE), ceramide (Cer) and triglycerides (TG).

### Performance of the lipidomic assay

Reproducibility of the lipidomic analysis was assessed by the incorporation of reference pooled plasma samples in the analysis sequence. The median percentage coefficient of variation (%CV) for the individual lipid species within the reference samples were 15.9%.

### Lipidomic phenotypes in relation to HLA haplotypes

**Fig. 2:**
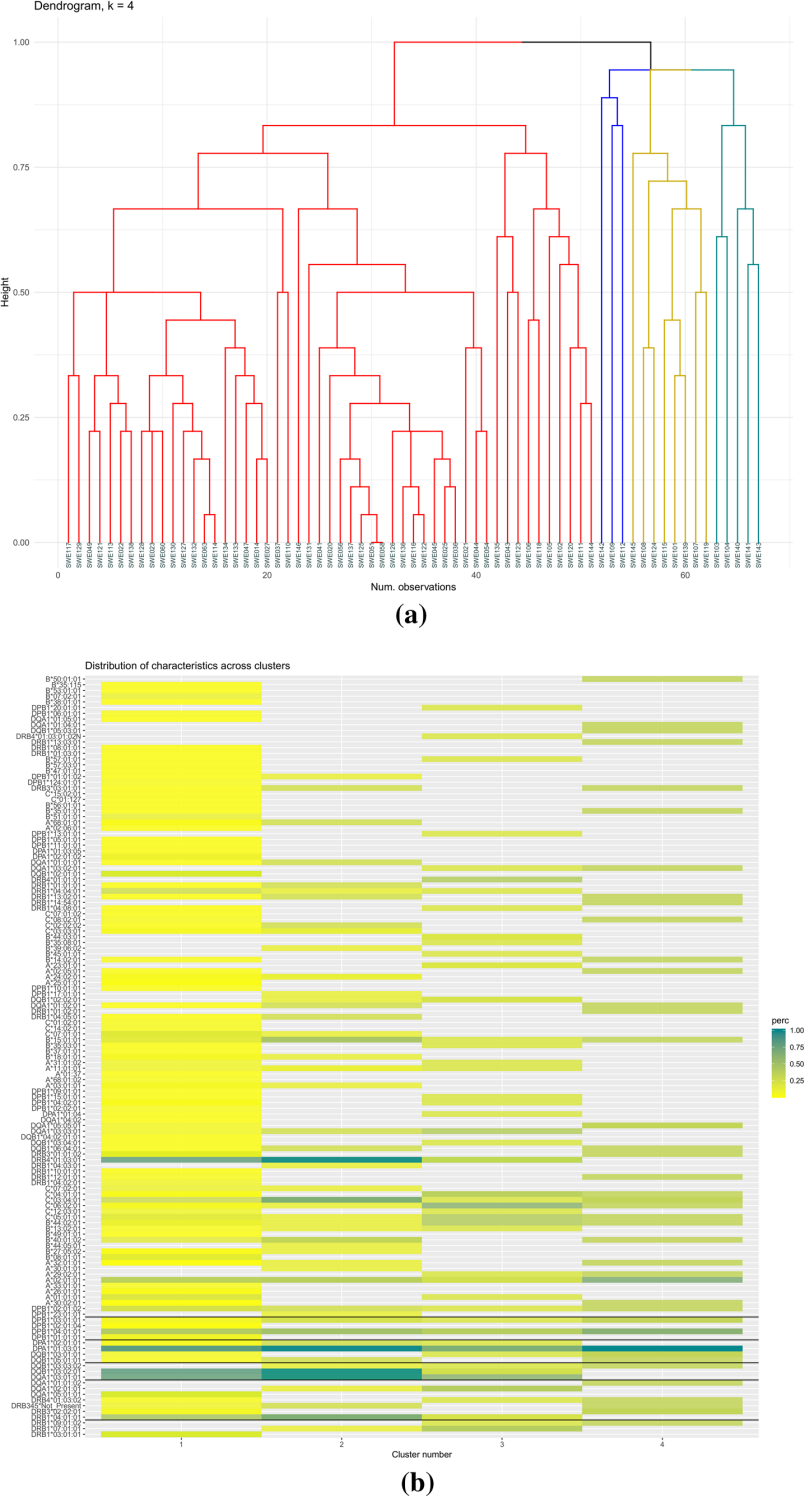

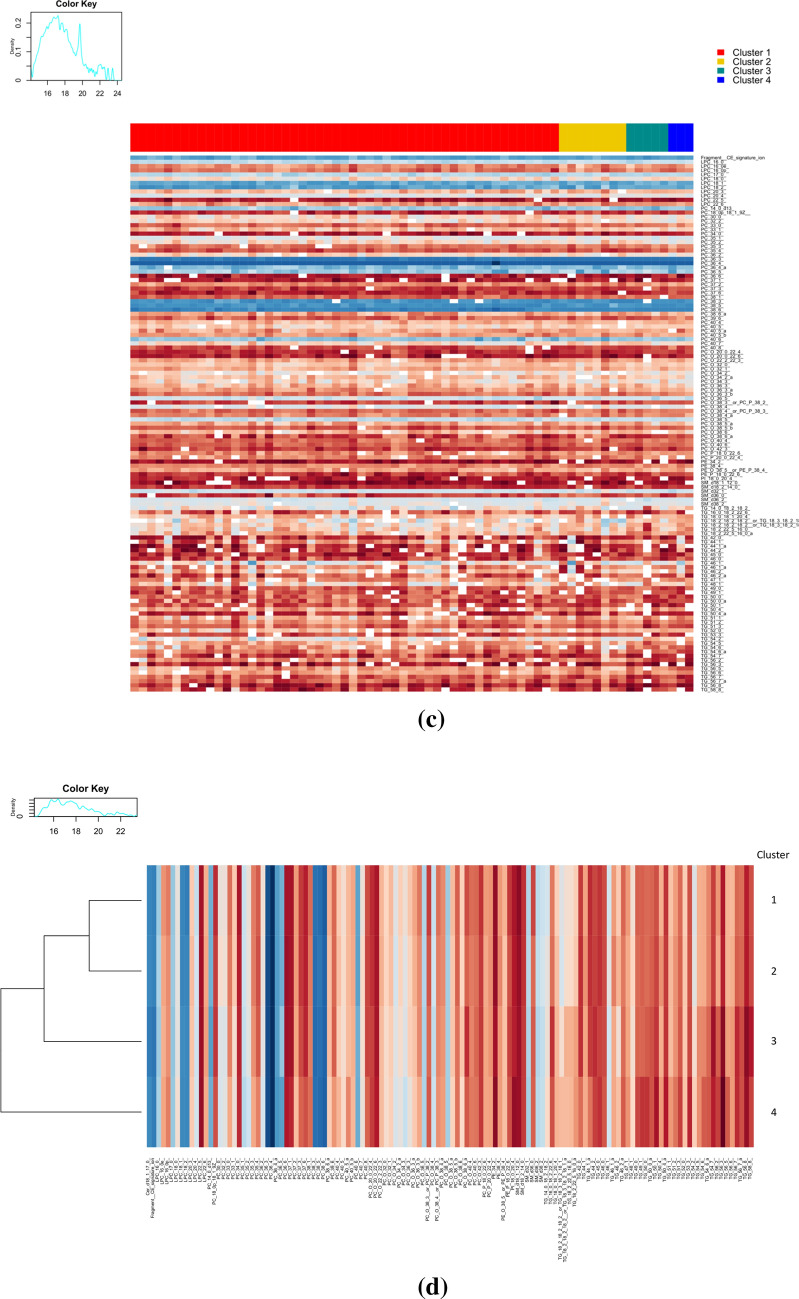
Hierarchical cluster analysis based on the HLA profiles and lipid variation between clusters. **a** Hierarchical cluster analysis based on the HLA profiles of the children. **b** The heatmap shows the absolute counts per variable across the four clusters based on the hierarchical cluster analysis of HLA profiles. The deeper green color corresponds to a higher number of observations within a cluster. The heatmap (**c**) and (**d**) shows the normalized lipid profiles adjusted for covariates (age, sex and autoimmunity burden measured as area under the curve of islet autoantibodies during DiPiS follow-up), and means of lipids, for each of the four HLA based clusters. The color-coded clusters are displayed on the top of the columns with individual id’s. Cluster 1 (in **b** and **d**) corresponds to red (in **a** and **c**). Cluster 2 (in **b** and **d**) corresponds to gold (in **a** and **c**). Cluster 3 (in **b** and **d**) corresponds to green (in **a** and **c**). Cluster 4 (in **b** and **d**) corresponds to blue (in **a** and **c**) (Color figure online)

In order to identify lipidomic specific phenotypes in relation to HLA haplotypes, we performed hierarchical cluster analysis to first group subjects based on their HLA profiles and haplotypes, followed by the generation of heat maps of lipid expression based on the observed clusters. To show how observations are distributed across categories; a colored dendrogram (Fig. 2a), a heatmap of observations count per variable within each cluster (Fig. 2b), a heat map lipid abundance within the clusters were created for visualization (Fig. 2c) and mean of lipid quantities based on the four generated clusters (Fig. 2d) were generated. The hierarchical cluster analysis identified four major clusters according to the HLA haplotypes. The largest cluster (Fig. 2a, red) includes many of the different haplotypes in the participants whereas the other three clusters (Fig. 2a, blue, green and gold) were defined by more specific haplotypes. The heat map of the mean value of the lipids within the clusters identifies cluster 3 as the most distinct cluster. This cluster was dominated by the presence of the DPB1*20:01:01, DRB4*01:03:01:02N, and DPB1*13:01:01 alleles in 50% of the participants and the DRB4*01:01:01 allele in 75% of the participants. Levels of PC (O-38:6) (a) were lower and PC (36:5) higher in this cluster compared to the other clusters. (Fig. 2d). Overall, the levels of TG (18:2/18:2/18:2) or TG (18:3/18:2/18:1) were decreasing from cluster 1–4 and several long chain TG were lower detected in cluster 4 [e.g., TG (50:1), TG (50:4a), TG (53:3 and TG (56:3)].

### Lipid differences according to autoantibody status

**Fig. 3:**
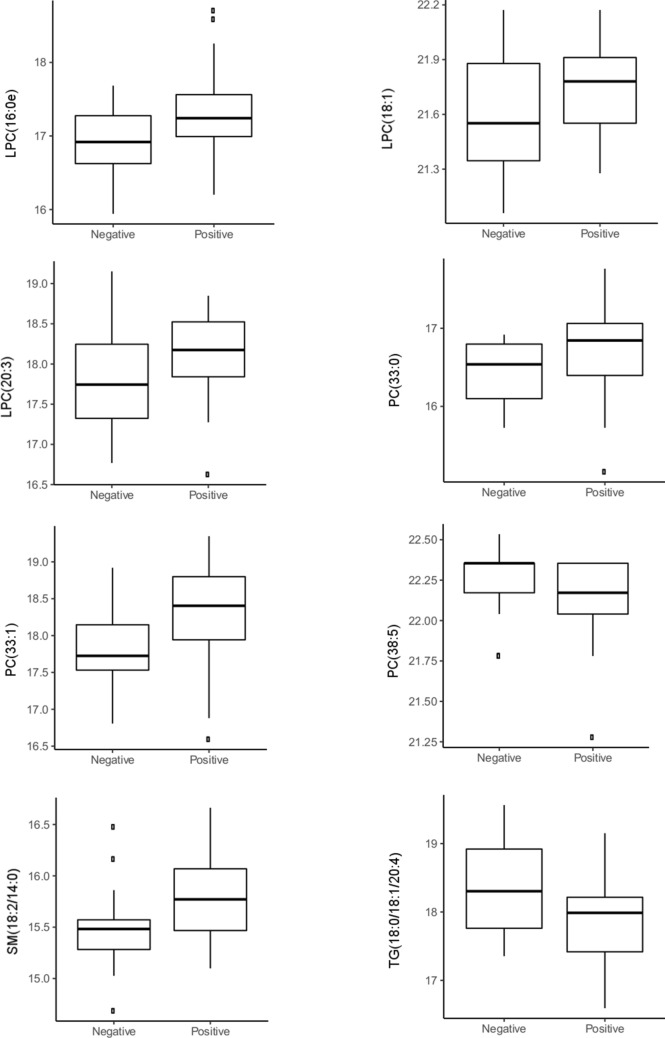
Boxplot of means of significantly expressed lipids and autoantibody status. The lipid data were corrected for age, sex and autoimmunity burden, measured as area under the curve of islet autoantibodies during DiPiS follow-up

Variation in the lipidome due to autoimmunity was assessed by dividing the subjects in two groups: negative for any autoantibody or positive for one or more autoantibodies (Fig. 3, boxplot of lipidomic variation and autoantibody status where lipids are corrected for age, sex and autoimmunity burden measured as area under the curve of autoantibodies during DiPiS follow-up). Three LPCs and two PCs were detected in significant higher levels in children positive for autoantibodies: LPC (16:0e) p_corr_ = 0.016 (95th CI 0.7–2.6), LPC (18:1) p_corr_ = 0.033 (95th CI 0.8–3.9), LPC (20:3) p_corr_ = 0.049 (95th CI 0.3–2), PC (33:0) p_corr_ = 0.042, (95th CI 0.4–2.4), PC (33:1) p_corr_ = 0.003 (95th CI 0.8–2.6). A single SM was detected in higher levels in autoantibody positive research subjects: SM (d18:2/14:0) p_corr_ = 0.013 (95th CI 0.9–3.6). One PC and one TG were detected in lower amounts in subjects positive for autoantibodies: PC (38:5) p_corr_ = 0.046 (95th CI − 6.6 to − 1.2) and TG (18:0/18:1/20:4) p_corr_ = 0.035, (95th CI − 1.8 to − 0.4). Supplementary Table 1 list intersects, CI, p-value and BH adjusted p-value for all lipids between groups.

### Levels of glucosylated ceramides (GlcCer) in relation to specific autoantibodies

**Fig. 4:**
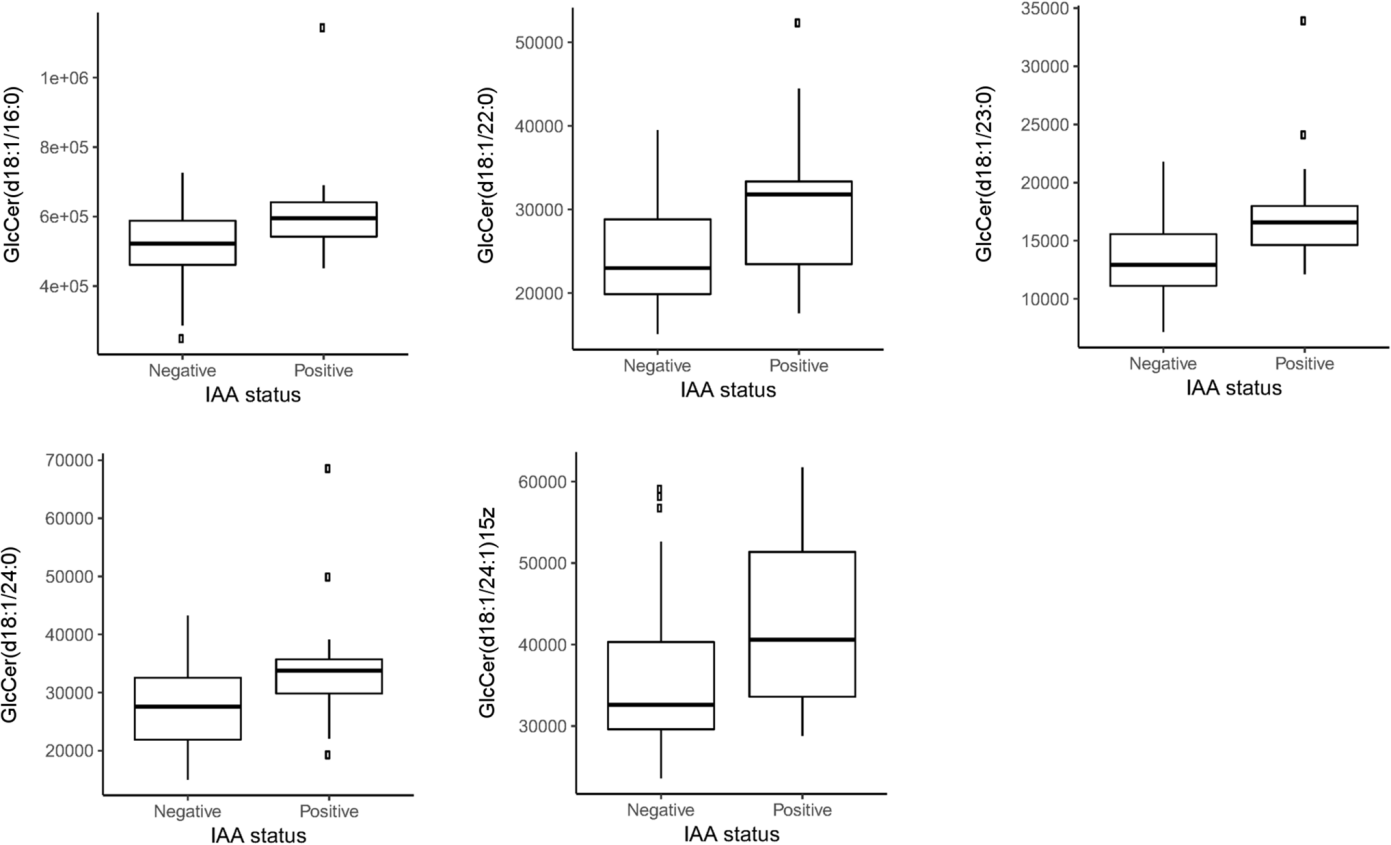
Boxplot of means for levels of ceramides and IAA status. ANOVA analysis of IAA status and five ceramides (LipidMaps ID) were significant (p < 0.05) in the DiPiS cohort (positive for IAA and other autoantibodies, n = 3 singly positive for IAA). Associations are corrected for age and gender. Boxplot of mean

We further investigated five glucosylated ceramides GlcCer (18:0/16:0), GlcCer (18:0/22:0), GlcCer (18:0/23:0), GlcCer (18:0/24:0) and GlcCer (18:0/24:1), which differed between groups, positive or negative for IAA. GlcCer were identified in spectres based on the online LipidMaps database. Individuals who were positive for IAA, independent of other autoantibodies, had significantly higher levels of the five GlcCer (Fig. 4). P values and confidence intervals of the five GlcCer are: GlcCer (18:1/16:0) p = 0.004, CI (5–95%): (3.35E+04 to 1.64E+05), GlcCer (18:1/22:0) p = 0.0007, CI (5–95%): (2.71E+03 to 1.01E+04), GlcCer (18:1/23:0) p = 0.0009, CI (5–95%): (1.81E+03 to 6.64E+03), GlcCer (18:1/24:0) p = 0.002, CI (5–95%): (2.45E+03 to 1.12E+04) and GlcCer (18:1/24:1) p = 0.005, CI (5–95%): (2.17E+03 to 1.21E+04). No correlations with any of the other autoantibodies were observed.

## Discussion

Variation in blood lipids in adolescent subjects, 10–15 years of age, with increased genetic risk for T1D and different stages of autoimmunity were investigated in this study. Previous analysis of the DiPiS cohort characterized lipidomic phenotypes in DiPiS subjects who developed diabetes before 8 years of age (La Torre et al. 2013). The DiPiS subjects in the present study had developed no, one or multiple islet autoantibodies but had not yet been diagnosed with T1D at time of blood sampling. Five subjects positive for one or multiple autoantibodies were diagnosed with T1D 6 to 26 months after blood sampling.

There is a lack in understanding the triggering and progression of the autoimmune destruction of the beta cells in T1D. An association between HLA and the risk of a first autoantibody have been suggested to be primary to an association between HLA and T1D (Elding Larsson et al. 2014; Honkanen et al. 2017; Ilonen et al. 2013; Krischer et al. 2015). Recent data support an association between HLA-DR-DQ and the first appearing autoantibody (Elding Larsson et al. 2014; Ilonen et al. 2013; Krischer et al. 2015), and that the first-appearing autoantibody may be associated with the age at onset of clinical T1D.

The variation in HLA haplotypes made it challenging to dissect if lipidomic profiles were associated with specific HLA types. However, hierarchical cluster analysis enabled comparison of lipids with clusters of HLA and detected lower and higher levels respectively of PC (O-38:6) (a) and PC (36:5) in cluster 3 defined by the presence of the DPB1*20:01:01, DRB4*01:03:01:02N, and DPB1*13:01:01 alleles. In addition, hierarchical cluster analysis demonstrated a tendency to lower levels of long-chain TGs in cluster three and four compared to the other clusters, which were high risk HLA-DQ2/8 (cluster 1) and non-HLA-DQ2/8 with homozygous or heterozygous DQ8. In cluster three, all were non-HLA-DQ2/8 and in cluster four three of the subjects were heterozygous for DQ8. It is well known that certain HLA haplotypes confer a high risk for developing T1D and also that increasing number of autoantibodies increase the risk for progression to clinical onset (Krischer et al. 2019; Ziegler et al. 2013). If, in the future HLA and autoantibodies can be matched with variations in lipid levels, lipidomic profiling could potentially be a novel way to improve prediction, and monitor disease progression.

In the present study we investigated variation in the lipidome due to autoimmunity (positive or negative for autoantibodies) and not number of autoantibodies, due to the cohort size and the size of our groups (Table 1). By analyzing lipid variation according to autoantibody status, three LPCs, two PCs, and a single SM were detected in significantly higher levels in subjects positive for autoantibodies. One PC and a TG were detected in lower amounts in subjects positive for autoantibodies. LPC, TGs and SMs have previously been associated to development of T1D in the DiPiS cohort (Oresic et al. 2013) in early childhood (< 3.6 years). Our results indicate that variation in phospholipids are related to autoimmunity and not necessarily progression to T1D, since we did not see any differences in the five subjects that developed T1D after end of follow-up.

Lamichhane et al. (2019) identified minor differences in levels of PCs and PEs in 3 months old children progressing to autoantibody positivity, but not T1D, in the follow up period until the age of 3 years. Our results shows that these differences in phospholipids also can be seen in healthy individuals, coming into adolescence, that are autoantibody positive. Our results support that some variation in phospholipids are a reflection of autoimmunity rather than rapid progression to T1D.

Early immune developmental processes in T1D progressors have in previous studies been suggested to be disturbed by distinct cord blood phospholipids and TGs. Also, a characteristic lipidomic profile has been found to be present already at birth in T1D progressors (La Torre et al. 2013; Oresic et al. 2013). We detected variation in only one TG species supporting the hypothesis raised by Oresic and colleagues regarding low TG levels, perhaps combined with short gestational age, as a risk factor limited to T1D diagnosis in very early childhood (< 2 years) (La Torre et al. 2013).

In previous studies it has also been suggested that SMs, as well as TGs, are potent regulators of immunogenic processes and play a potent role in inflammatory disease (Iannello et al. 2003; Olivera and Rivera 2005). However, we did not find any differences in lipid levels when comparing the five subjects who developed T1D after sampling to those who did not.

Significantly higher levels of GlcCer were found in the group of IAA positive subjects. As IAA are often observed in children developing T1D in early childhood, we speculate that this is an aggressive form of T1D pathogenesis that may be reflected in these lipids. It has been shown that ceramide plays a significant role in diabetes by inducing β-cell apoptosis, modulate insulin signaling and causing insulin resistance (Galadari et al. 2013). Our analysis disregard by which autoantibody autoimmunity was first initiated however, our analysis identify an interesting association between GlcCer levels and IAA positivity. GlcCer is a group of lipids previously shown to associate to ER stress, cardiovascular disease and diabetes (Boslem et al. 2011; Chavez et al. 2014; Messner and Cabot 2010) and has been proposed to have pro-apoptotic effects (Pettus et al. 2002). Recent studies have shown that children representing autoimmunity with positivity for IAA first, have a faster, more severe, progression to T1D (Krischer et al. 2019). Future studies should include investigation of GlcCer levels in individuals exclusively positive for IAA.

The main strengths of the present study are the long term follow up of subjects randomly selected from the DiPiS study, deep HLA sequencing that enabled determination of exact HLA genotypes as well as the detailed lipidomic profiling. None of the subjects had diabetes at the time of sampling. All of the subjects have an elevated risk for T1D and have been followed in the DiPiS study since 2 years of age. The follow-up in DiPiS was not critical for the lipidomics analysis but enabled detailed interrogation of the development of autoantibodies prior to cross-sectional sampling, in relation to HLA haplotypes.

At the time of sampling, 13 subjects had never had autoantibodies at any times during DiPiS follow-up. We expected to have autoantibody negative subjects but did not expect that all would have the highest HLA risk (HLA-DQ2/8). At the same time, the HLA-DQ2/8 genotype is underrepresented in the groups of one and multiple autoantibodies, perhaps because these subjects would already have progressed to clinical T1D. We speculate that the autoantibody negative subjects may not develop T1D, maybe due to lack of trigger event. A close look at the lipidomic profiles of the five subjects that progressed to T1D after cross-sectional sampling did not reveal any significant difference in comparison to the autoantibody-positive subjects that to date have not developed T1D indicating that the variation we detect in phospholipids could be related primarily to autoimmunity. However caution should be made when concluding on these results, because of the low numbers of progressors to T1D.

A weakness of the study would be the limited number of participants that makes it difficult to generalize since many different HLA haplotypes are represented and cellular analyses were not available during follow up. The present study cohort size and distribution between groups would have made it difficult to interpret and generalize any results from comparing the groups presented in Table 1, instead of autoimmunity as in the present study. Therefore, it would be of interest for future studies to analyze autoantibodies between groups of research subjects without and with one or multiple autoantibodies. We speculate that this type of analysis would increase the knowledge of lipids levels and if they tend to vary with increasing number of autoantibodies.

Another limitation is that the subjects were not fasting before blood draw. It was not possible to have the subjects fasting before showing up for blood draw, since many of the subjects had to travel far to get to the study center. Previous published data show little direct effect on test meal fatty acid composition and post prandial lipid composition of the blood (Jackson et al. 1999) and furthermore, a long-term twin study showing high heritability of particularly phospholipids, irrespective of a 5-week dietary intervention (Frahnow et al. 2017) and so we felt confident in using these samples regardless. However, in future clinical association studies, we aim to standardize diet in our subjects.

## Conclusion

Variation in blood lipids in adolescent subjects with an increased genetic risk for T1D and different stages of autoimmunity were investigated. The lipidomic profiling in this study provides insight into the lipid composition in adolescents who had developed islet autoimmunity and are at an increased risk for T1D. Our findings support the hypothesis and previous findings that lipids may vary with islet autoimmunity. Future investigations of the observed tendencies that lipidomic profiles may be associated with HLA will be necessary. Future studies will have to confirm the value of these profiles and if the findings can be used as early markers for T1D. Further studies of lipidomics to clarify the potential role of lipids in the risk for type 1 diabetes are warranted.

## Electronic supplementary material

Below is the link to the electronic supplementary material.

(TIF 853 kb)

## Data Availability

The lipidomic data reported in this paper are available via https://figshare.com/ and study identifier 10.6084/m9.figshare.11341778.

## Abbreviations

DiPiS: Diabetes Prediction in Skåne
CE: Cholesterol ester
Cer: Ceramide
GADA: Glutamate decarboxylase autoantibody
GlcCer: Glucosylated ceramides
HLA: Human leukocyte antigen
IA-2A: Insulinoma-associated protein-2 autoantibody
IAA: Insulin autoantibody
IASP: Islet Autoantibody Standardization Program
LPC: Lysophosphatidylcholine
PA: Phosphatic acid
PC: Phosphatidylcholine
PC(O): Alkylphosphatidylcholine
PC(P): Plasmalogen, alkenylphosphatidylcholine
PE: Phosphatidylethanolamine
PE(O): Alkylphosphatidylethanolamine
PE(P): Phosphatidylethanolamine plasmalogen
PI: Phosphatidylinositol
PG: Phosphatidylglycerol
PS: Phosphatidylserine
SM: Sphingomyelin
T1D: Type 1 diabetes
TG: Triacylglycerol
ZnT8A: Zinc Transporter 8 autoantibody
