# Supplementary figures and images for: Characterization of plasma lipidomics in adolescent subjects with increased risk for type 1 diabetes in the DiPiS cohort
Source: Metabolomics. 2020 Oct 8;16(10):109. doi: 10.1007/s11306-020-01730-x (PMC7544716; doi:10.1007/s11306-020-01730-x)

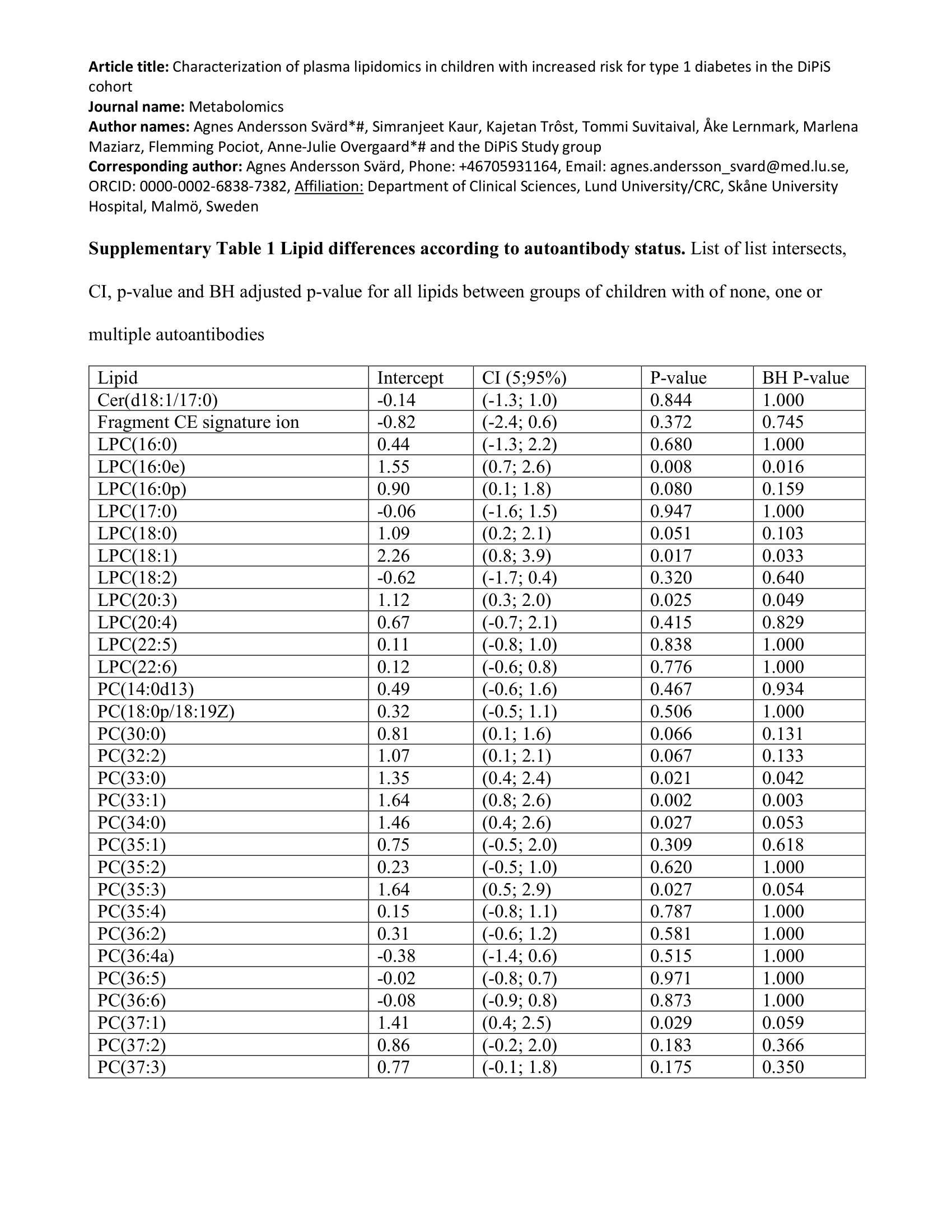

Supplement: Supplementary file 1 — (TIF 853 kb) [file 11306_2020_1730_MOESM1_ESM.tif]
